# Anatomical features of the incisivus labii superioris muscle and its relationships with the upper mucolabial fold, labial glands, and modiolar area

**DOI:** 10.1038/s41598-018-31334-4

**Published:** 2018-08-27

**Authors:** Mi-Sun Hur

**Affiliations:** Department of Anatomy, Catholic Kwandong University College of Medicine, Gangneung, Gangwon-do 25601 Republic of Korea

## Abstract

The current study examined the incisivus labii superioris muscle (ILS) and its morphologic and spatial relationships with the surrounding structures, especially focusing on the upper mucolabial fold, labial glands, and modiolar area. ILSs were investigated in 52 specimens obtained from formalin-fixed Korean adult cadavers (26 left sides, 26 right sides of the face; 15 men, 11 women; mean age, 70.8 years). ILSs were observed in all specimens (100%). The ILS has an oblique and linear origin from the incisive fossa of the maxilla to the point just medial to the origin of the levator anguli oris muscle (LAO). The arising fibers of the ILS arched and covered the prominent labial glands at the superior margin of the orbicularis oris muscle (OOr). After the ILS coursed laterally along the anterior part of the upper mucolabial fold, it divided into superficial and deep inserting fibers in 48 specimens (92.3%) and it did not divide in 4 specimens (7.7%). The superficial inserting ILS fibers and the ILS fibers that did not divide blended with the medial fibers of the LAO to converge toward the modiolus. The deep inserting fibers of the ILS blended with the lateral deep fibers of the OOr in the 48 specimens (92.3%), and the deep inserting fibers continued to descend to converge toward the modiolus in 20 of those specimens (38.5%). These observations indicate that the ILS may assist to compress the labial glands and the upper oral vestibule, controlling modiolar movements and thereby integrating the movements of the mouth and lips.

## Introduction

The orbicularis oris muscle (OOr) is a complex sphincter muscle consisting of numerous strata of muscular fibers surrounding the orifice of the mouth but orientated in different directions^1^. An ultrasound study found that although the OOr may be regarded as a single muscle anatomically, it appears functionally to consist of different parts acting either independently or together with other facial muscles^2^. The incisivus labii superioris muscle (ILS) comprises the fibers of the OOr that are connected to the maxilla^1^. The ILS and incisivus labii inferioris muscle (ILI) draw the corners of the lips medially, and the OOr draws the upper lip downward. The circumferential portion of the OOr acts with the ILS and ILI to make the lips protrude^3^.

The ILS reportedly arises from the incisive fossa of the maxilla and arches laterally, interlacing with fibers of the peripheral part of the OOr. The ILS partially blends with the levator anguli oris muscle (LAO) and attaches to the modiolus^4,5^. The OOr and the modiolus, which are insertions of the ILS, are positioned by their attaching muscles. The most obvious determinant of the position and mobility of the modiolus is the net force exerted by the muscles that are directly attached to it^5^. The OOr has no bony or cartilaginous attachment^6,7^, and so this muscle can be drawn toward the contracting muscle that converges to the OOr. Understanding and analyzing the movements of the mouth and lips requires knowledge of how the ILS courses and attaches to other structures near the oral vestibule and in the modiolar area.

The interlacing of the bundles of the various muscles at the corner of the mouth has evolutionarily become more complex in humans^8^, with the degree of differentiation of the ILS being greater in humans than in lower animals^9^. Burkitt and Lightoller (1926)^10^ described that the ILS is well developed and that its fibers are of the same size and pigmentation as in the OOr in Aboriginal Australians. There have been several reports about the morphology of the ILI and its anatomical relationships with surrounding structures in humans^11,12^. However, there have been few reports about the anatomy of the ILS^13^. The anatomical relationships of the ILS with the upper mucolabial fold, labial glands, and modiolar area therefore remain unclear.

The current study examined the ILS and its morphologic and spatial relationships with the surrounding structures, especially focusing on the upper mucolabial fold, labial glands, and modiolar area in order to improve the understanding of integrated activities involving these structures during movements of the mouth and lips. This information will also be helpful when performing various types of facial surgery including vestibuloplasty and cleft lip repair.

## Results

### Arising pattern and location of the ILS at its origin site

**Figure 1:**
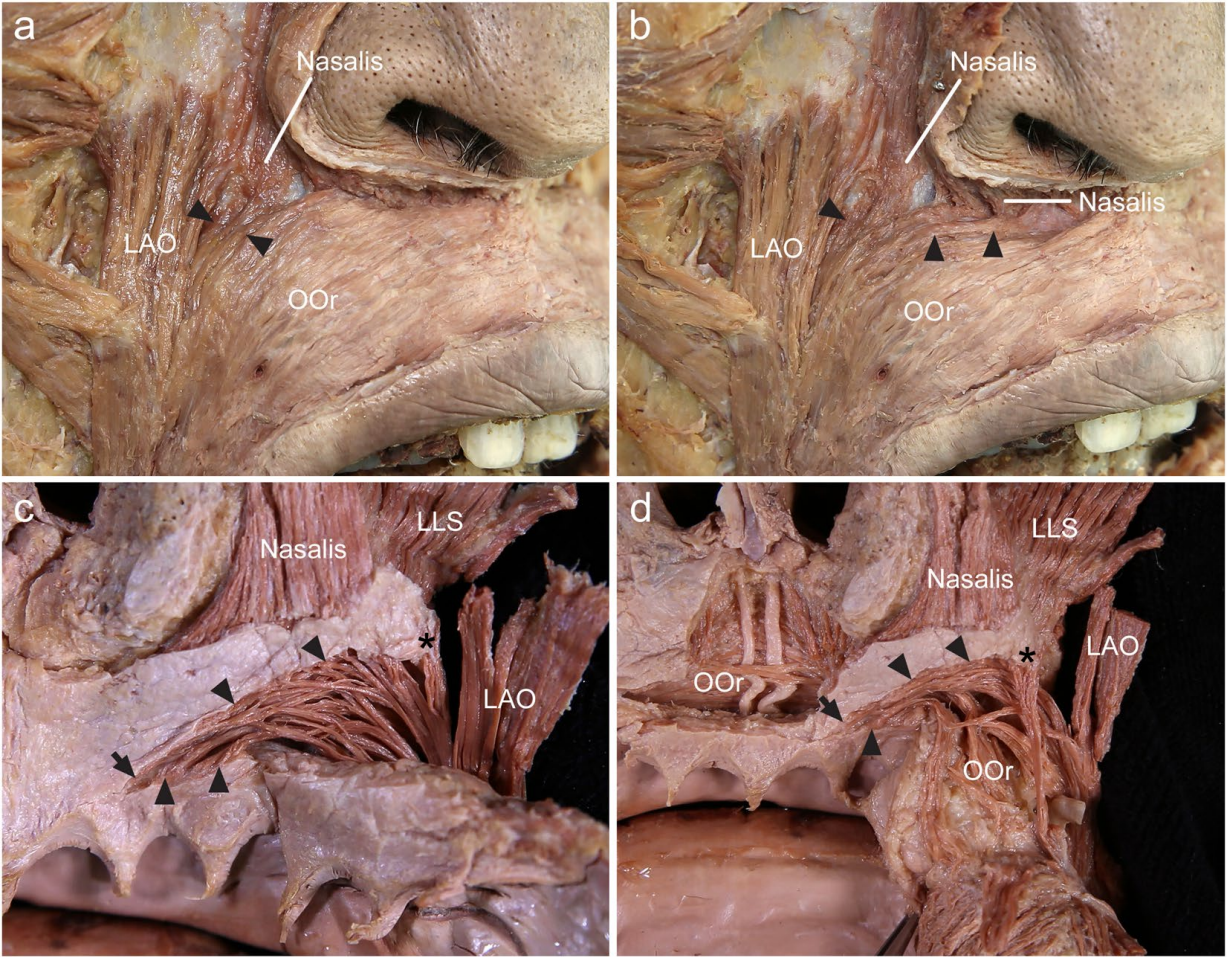
The arising fibers of the incisivus labii superioris muscle (ILS; arrowheads) at its origin site. (**a**) In the anterior aspect, the superior peripheral part of the orbicularis oris muscle (OOr) covered the medial arising fibers of the ILS. The lateral arising fibers of the ILS were not covered by the OOr, and these fibers were located between the OOr and levator anguli oris muscle (LAO). (**b**) In the anterior aspect, the ILS arose from the periosteum of the maxilla from the incisive fossa to the point just medial to the origin of the LAO. The arising fibers of the ILS were interdigitated with the arising fibers of the nasalis. The medial arising fibers arched laterally, while the lateral arising fibers descended almost straight downward. To reveal the attachment of the ILS to the maxilla, some fibers of the superior peripheral part of the OOr were pulled down. (**c**) In the posterior aspect, the ILS had an oblique and linear origin. The most-lateral point (asterisk) of origin of the ILS was located higher than its most-medial point (arrow) of origin. The periosteum adjacent to the upper mucolabial fold was cut and removed in order to reveal the origin of the ILS. (**d**) In the posterior aspect, the medial arising fibers of the ILS were located beneath the superior peripheral part of the OOr. As the course of the ILS arched laterally it became the superolateral margin of the OOr, enlarging the dimension of the superior peripheral part of the OOr. It deepened the superolateral margin of the OOr posteriorly. LLS, levator labii superioris muscle.

ILSs were observed in all specimens (100%). The ILS had an oblique and linear origin from the incisive fossa of the maxilla to the point just medial to the origin of the LAO. The most-lateral point of origin of the ILS was higher than its most-medial point of origin. The most-medial point of origin of the ILS was located in the periosteum adjacent to the upper mucolabial fold. The majority of the medial origin of the ILS was located in the periosteum just below the upper mucolabial fold, and the minority was located in the periosteum just above the upper mucolabial fold. The origin of the ILS between the most-medial point and the most-lateral point was located in the periosteum where the transverse and alar parts of the nasalis arose. The most-lateral point of the ILS was located just medial to the origin of the LAO. The medially arising fibers of the ILS coursed deep to the superior peripheral part of the OOr and these fibers curved upward and laterally. The medial two-thirds of the arising fibers were usually more curved than the lateral one-third of the fibers. The lateral one-third of the arising fibers descended almost straight downward. As the course of the ILS arched laterally it became the superolateral margin of the OOr, enlarging the dimension of the superior peripheral part of the OOr. It also deepened the superolateral margin of the OOr posteriorly (Fig. 1).

**Figure 2:**
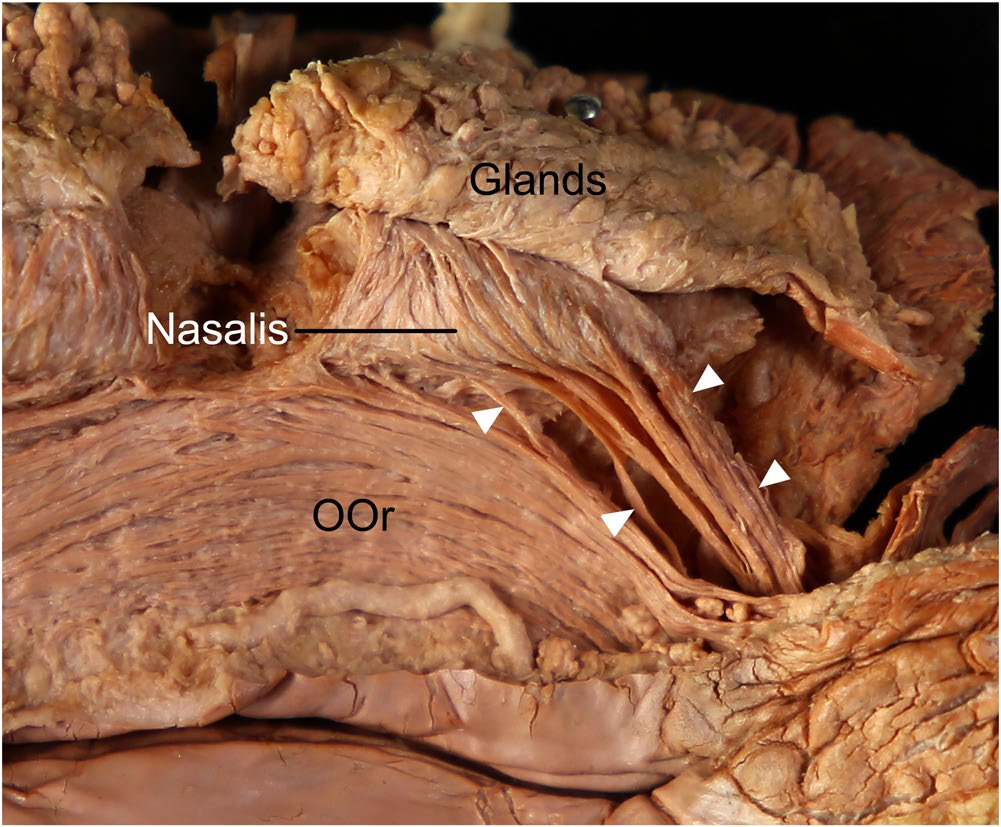
The arising fibers of the incisivus labii superioris muscle (ILS; arrowheads) at its origin site in the posterior aspect. The arising fibers of the ILS coursed just anterior to those of the transverse and alar parts of the nasalis. Some arising fibers of the ILS were interlaced with those of the transverse and alar parts of the nasalis. To reveal the origin of the ILS, the periosteum adjacent to the upper mucolabial fold was cut and then reflected superiorly with its attached fibers of the ILS, nasalis, and labial glands. OOr, orbicularis oris muscle.

The origin of the ILS usually coincided with that of the transverse and alar parts of the nasalis. The arising fibers of the ILS coursed just anterior to those of the transverse and alar parts of the nasalis. Some arising fibers of the ILS were interdigitated at its origin site with those of the transverse and alar parts of the nasalis. The arising fibers of the ILS coursed laterally, while those of the transverse and alar parts of the nasalis coursed upward (Fig. 2).

**Figure 3:**
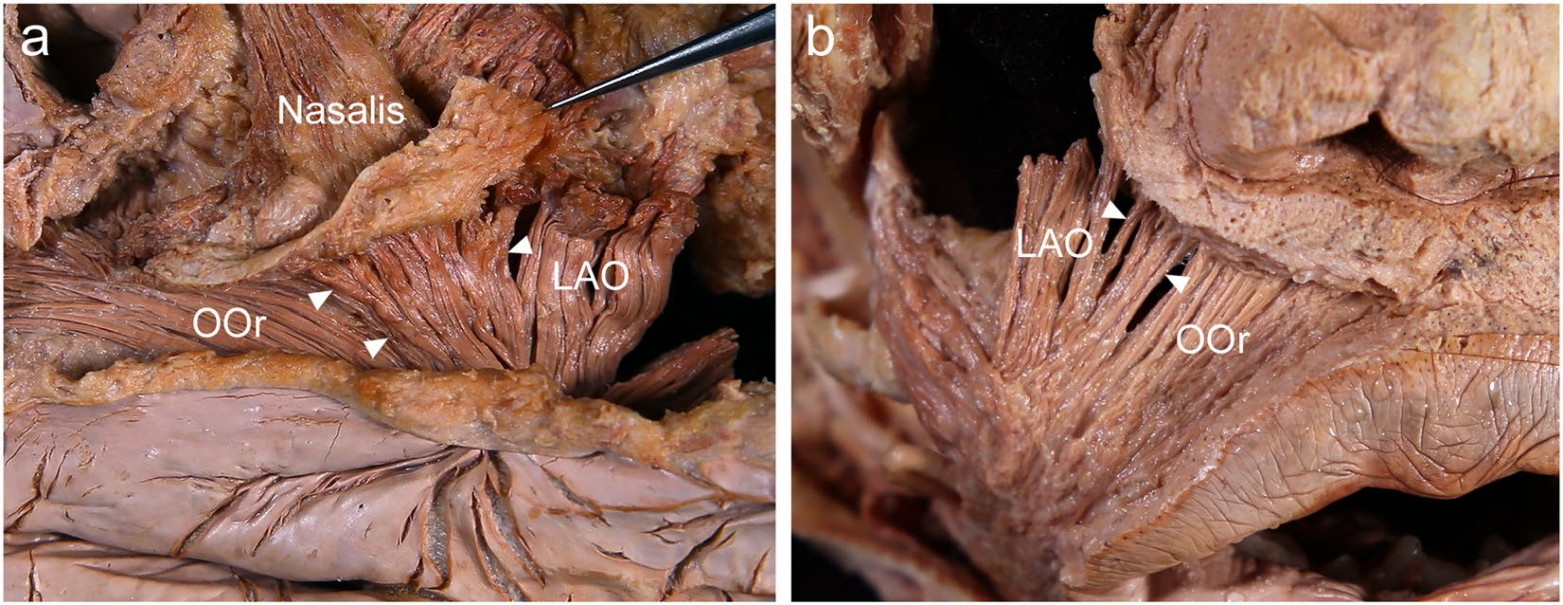
Continuous arrangement of the incisivus labii superioris muscle (ILS; arrowheads) between the orbicularis oris muscle (OOr) and the levator anguli oris muscle (LAO). The ILS was located (**a**) between the OOr and LAO in a fan shape in the deep surface and (**b**) on the superficial surface of the face. To reveal the ILS, the periosteum adjacent to the upper mucolabial fold was cut and then reflected superiorly with its attached fibers of the ILS.

The ILS muscle fibers were located between the OOr and LAO in a fan shape. In addition, the medially arising fibers of the LAO usually curved slightly toward the ILS. Thus, the OOr, ILS, and LAO were arranged in a continuous pattern (Fig. 3).

### Location of origin of the ILS on the superficial surface of the face

**Figure 4:**
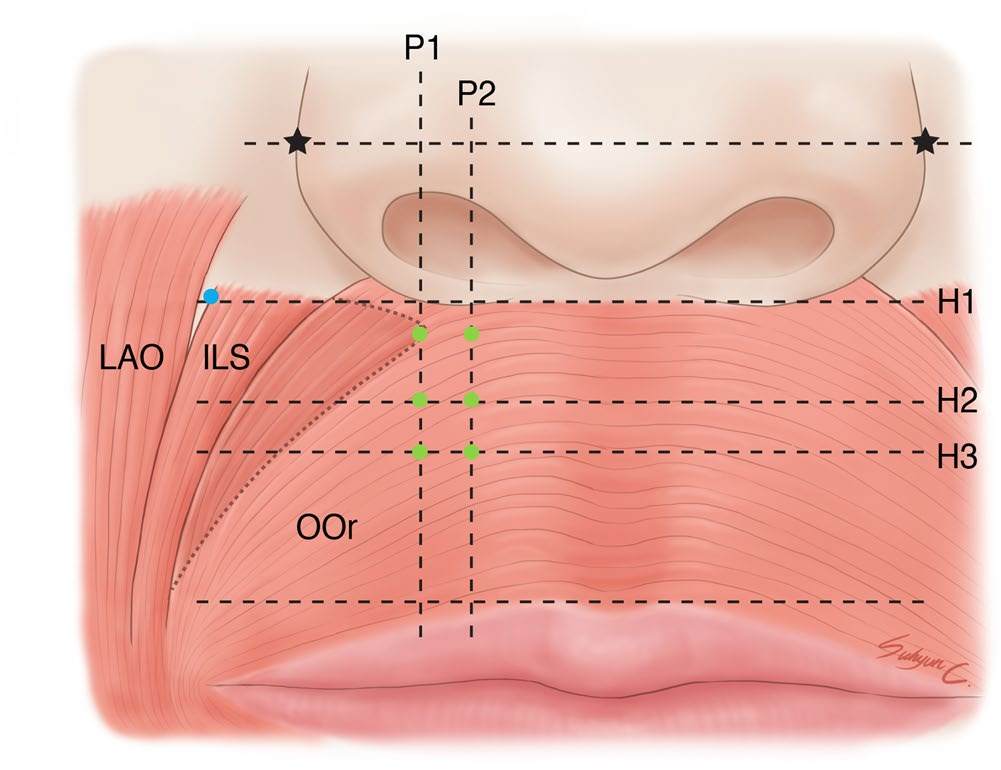
Locations of the origin of the incisivus labii superioris muscle (ILS) on the superficial surface of the face. Green dots indicate locations of the most-medial points of origin of the ILS, and the blue dot indicates the location of the most-lateral point of origin of the ILS. Asterisks indicate the alare. P1 and P2 indicate perpendicular lines passing through the lateral one-third and the middle of the nostril, respectively. H1 indicates a horizontal line drawn just below the nostril sill. H2 and H3 indicate the upper-one-third horizontal line and the middle horizontal line passing between the nostril sill and vermilion border of the upper lip, respectively. LAO, levator anguli oris muscle. *Illustrations licensed under CC BY 4*.*0*.

The most-lateral and most-medial points of origin of the ILS were observed with their corresponding points on the superficial surface of the face. The most-lateral point of origin of the ILS was usually located from approximately 0.5 to 1.5 cm inferolaterally to the alare. The location of the most-medial point of origin of the ILS varied somewhat between the specimens, as follows (Fig. 4):

1. Where a perpendicular line passing through the lateral one-third of the nostril crossed a horizontal line passing just below the nostril sill (*n* = 16, 30.8%).
2. Where a perpendicular line passing through the lateral one-third of the nostril crossed an upper-one-third horizontal line passing between the nostril sill and vermilion border of the upper lip (*n* = 4, 7.7%).
3. Where a perpendicular line passing through the lateral one-third of the nostril crossed a middle horizontal line passing between the nostril sill and the vermilion border (*n* = 12, 23.1%).
4. Where a perpendicular line passing through the middle of the nostril crossed a horizontal line just below the nostril sill (*n* = 6, 11.5%).
5. Where a perpendicular line passing through the middle of the nostril crossed an upper-one-third horizontal line passing between the nostril sill and vermilion border of the upper lip (*n* = 2, 3.8%).
6. Where a perpendicular line passing through the middle of the nostril crossed a middle horizontal line passing between the nostril sill and the vermilion border (*n* = 12, 23.1%).

### Positional relationships of the ILS with the upper mucolabial fold and labial glands

**Figure 5:**
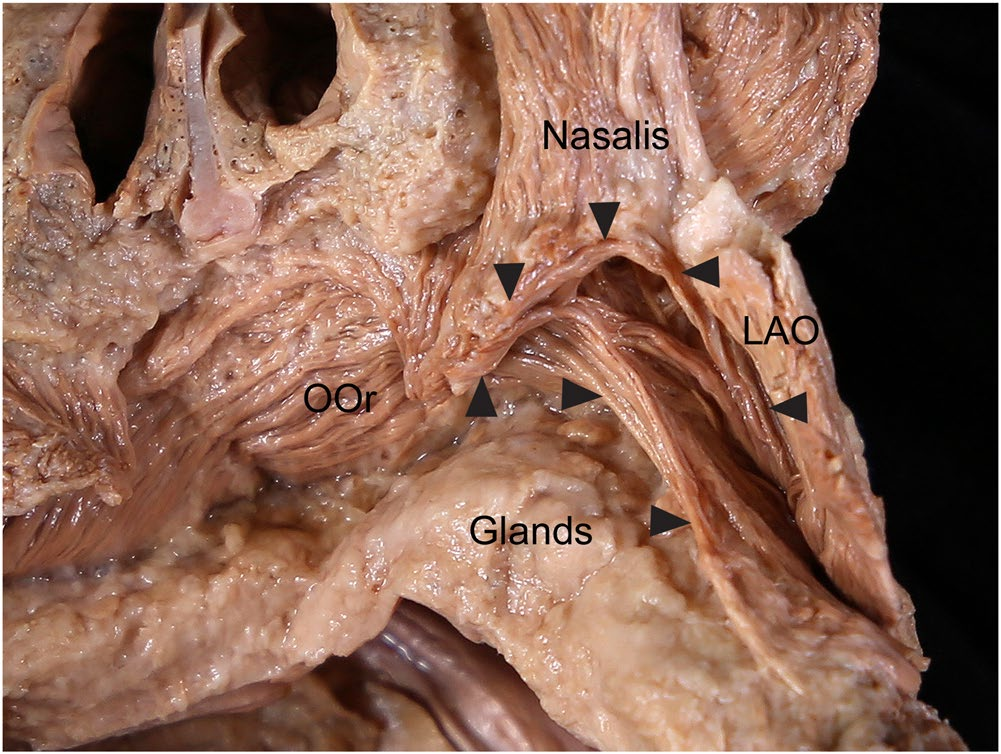
Spatial relationships of the incisivus labii superioris muscle (ILS; arrowheads) with the labial glands in the posterior aspect. The arising fibers of the ILS arched and covered the prominent labial glands at the superior margin of the orbicularis oris muscle (OOr). To show the ILS and the labial glands, the periosteum adjacent to the upper mucolabial fold was cut and the arising fibers of the ILS and the nasalis were elevated. LAO, levator anguli oris muscle.

The ILS coursed laterally just above the anterior part of the upper mucolabial fold. The upper mucolabial fold was located between the ILS and OOr in all specimens (100%). The arising fibers of the ILS arched and covered the prominent labial glands at the superior margin of the OOr (Fig. 5). The arched shape of arising fibers of the ILS coincided with the convex surface of the prominent labial glands. The arising fibers of the ILS were more arched when the labial glands were more prominent. In some cases a few fibers of the ILS blended with the superior fibers of the buccinator muscle just lateral to the OOr, and they covered a few labial glands located at the superior margin of the buccinator muscle.

### Insertion patterns of the ILS in the modiolar area

**Figure 6:**
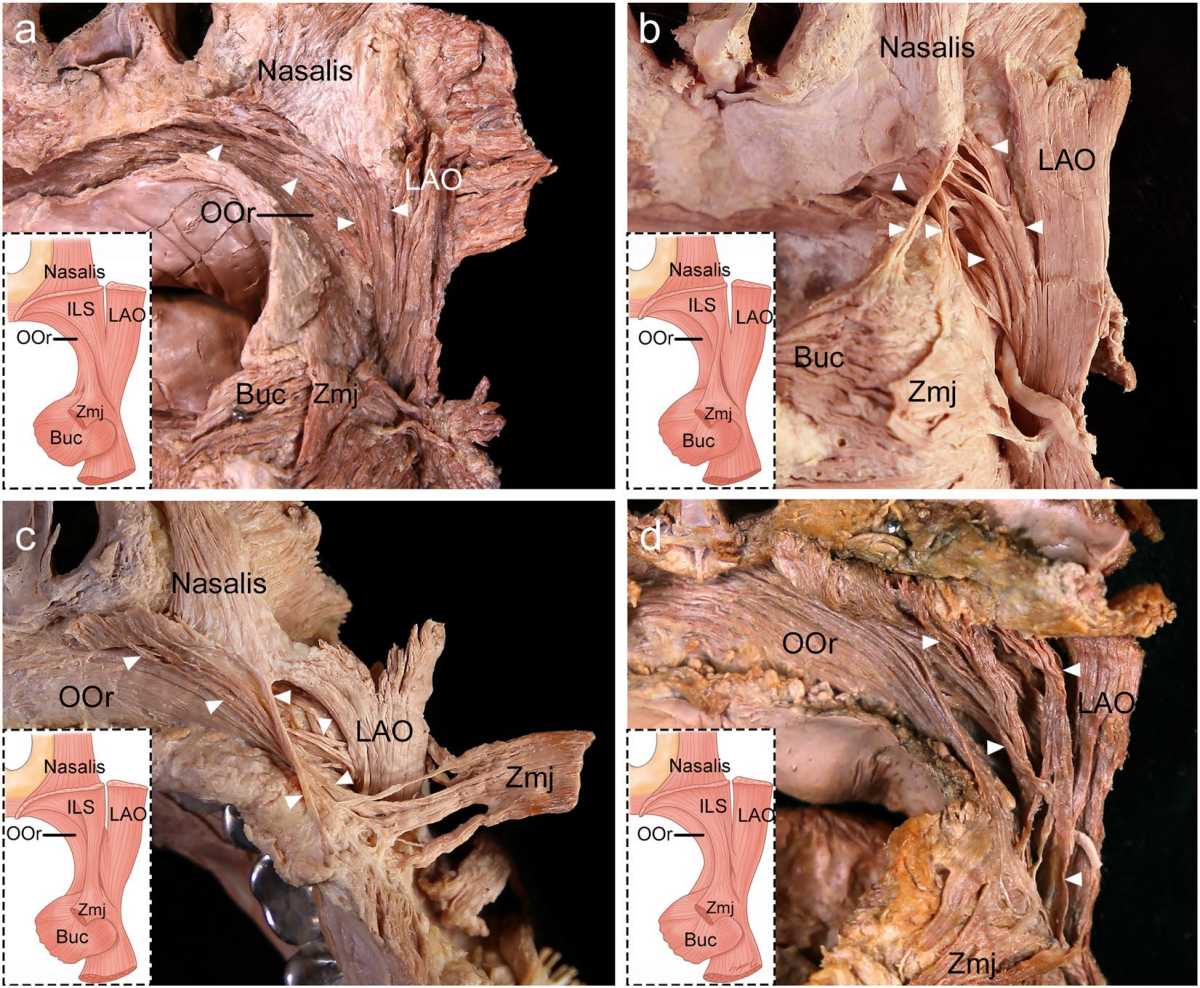
Four types of insertion patterns of the incisivus labii superioris muscle (ILS; arrowheads) in the modiolar area in the posterior aspect. (**a**) In type I, all of the ILS fibers blended with the medial fibers of the levator anguli oris muscle (LAO). (**b**) In type II, more than two-thirds of the ILS fibers became superficial inserting fibers to blend with the LAO; the other fibers became deep inserting fibers of the ILS. (**c**) In type III, approximately half of the ILS fibers became superficial inserting fibers and approximately half became deep inserting fibers. The superficial inserting fibers blended with the LAO. (**d**) In type IV, less than one-third of the ILS fibers became superficial inserting fibers to blend with the LAO, and the other fibers became deep inserting fibers of the ILS. The deep inserting fibers of the ILS blended with the buccinator muscle (Buc), the deep fibers of the zygomaticus major muscle (Zmj) at its insertion site, and the deep fibers of the orbicularis oris muscle (OOr). To show the arising fibers of the ILS, the periosteum adjacent to the upper mucolabial fold was cut and the arising fibers of the ILS and the nasalis were elevated. *Illustrations licensed under CC BY 4*.*0*.

After the ILS coursed laterally along the anterior part of the upper mucolabial fold, it divided into superficial and deep inserting fibers in 48 specimens (92.3%) and it did not divide in 4 specimens (7.7%). The superficial inserting ILS fibers and the ILS fibers that did not divide blended with the medial fibers of the LAO to converge toward the modiolus. The insertion patterns of the ILS fibers in the modiolar area could be classified into four types according to the number of ILS fibers that blended with the LAO (Fig. 6):

1. Type I (*n* = 4, 7.7%), in which all ILS fibers blended with the LAO.
2. Type II (*n* = 31, 59.6%), in which more than two-thirds of the ILS fibers became superficial inserting fibers to blend with the LAO; the other fibers became deep inserting fibers of the ILS. The other fibers arose as the medial one-third of fibers, the medial one-fourth of fibers, a few medial and lateral fibers, or some lateral fibers of the ILS.
3. Type III (*n* = 7, 13.5%), in which approximately half of the ILS fibers became superficial inserting fibers and approximately half became deep inserting fibers. The superficial inserting ILS fibers blended with the LAO.
4. Type IV (*n* = 10, 19.2%), in which less than one-third of the ILS fibers became superficial inserting fibers to blend with the LAO, and the other fibers became deep inserting fibers.

### Deep inserting fibers of the ILS in the modiolar area

**Figure 7:**
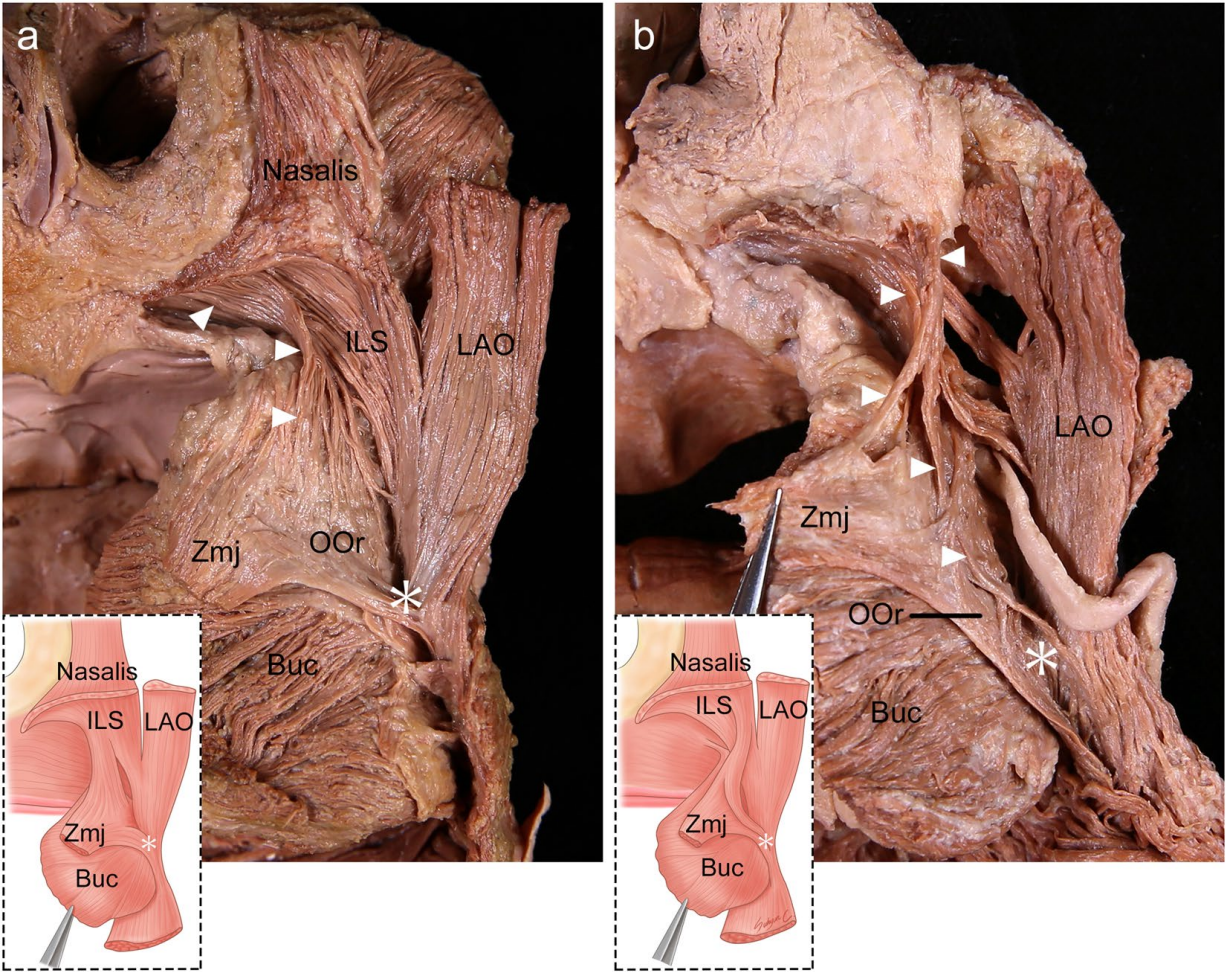
The deep inserting fibers of the incisivus labii superioris muscle (ILS; arrowheads) in the modiolar area in the posterior aspect. These fibers blended with the deep fibers of the orbicularis oris muscle (OOr) and deep fibers of the zygomaticus major muscle (Zmj) at its insertion site (**a**), and descended to converge toward the modiolus (**b**). To show the arising fibers of the ILS, the periosteum adjacent to the upper mucolabial fold was cut and the arising fibers of the ILS were elevated. Buc, buccinator muscle; LAO, levator anguli oris muscle. Asterisks indicate the modiolus. *Illustrations licensed under CC BY 4*.*0*.

The deep inserting fibers of the ILS blended with several muscles in the modiolar area: the deep fibers of the OOr, the deep fibers of the zygomaticus major muscle (Zmj), and sometimes the superior fibers of the buccinator muscle. The deep inserting fibers of the ILS blended with the lateral deep fibers of the OOr in the 48 specimens (92.3%), and the deep inserting fibers continued to descend to converge toward the modiolus in 20 of those specimens (38.5%) (Fig. 7). The modiolus was located below the level of the corner of the mouth in all specimens. Some of the ILS fibers were interdigitated or were connected to the deep fibers of the Zmj at or before its insertion site in 44 specimens (84.6%).

**Figure 8:**
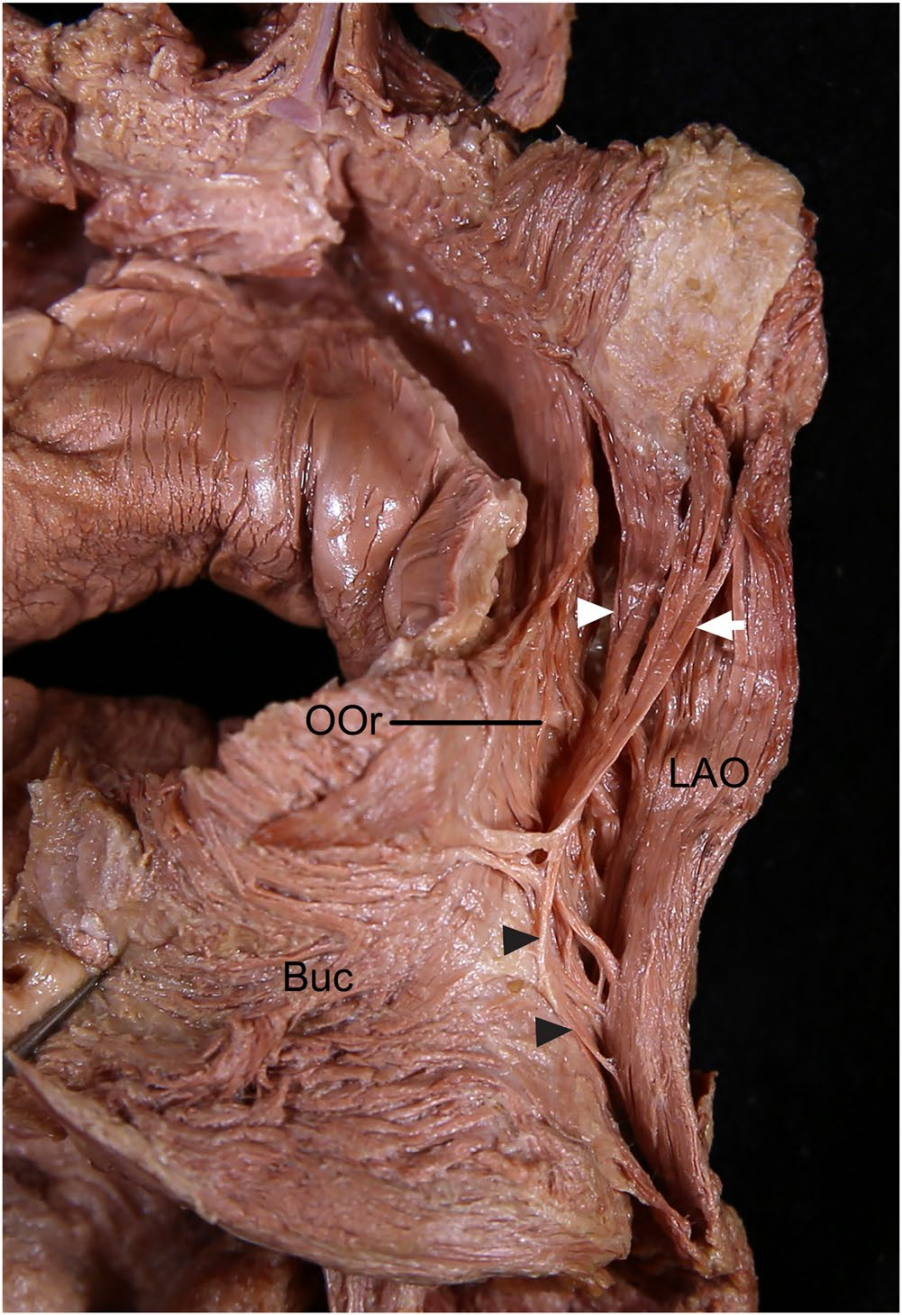
A thin muscle layer (black arrowheads) was formed from some fibers of the incisivus labii superioris muscle (ILS; white arrowhead) and the levator anguli oris muscle (LAO; arrow) in the posterior aspect. This thin muscle layer blended with the deep fibers of the orbicularis oris muscle (OOr) to converge toward the modiolus. To show the arising fibers of the ILS, the periosteum adjacent to the upper mucolabial fold was cut and the arising fibers of the ILS were elevated. Buc, buccinator muscle.

In 10 specimens (19.2%), some deep inserting fibers of the ILS and some fibers of the LAO formed a thin muscle layer located deep to the LAO (Fig. 8). In two specimens (3.8%), only some fibers of the ILS formed the thin muscle layer. The number of ILS and LAO fibers forming the thin layer varied among the specimens. The thin layer blended with the deep fibers of the OOr to converge toward the modiolus.

## Discussion

**Figure 9:**
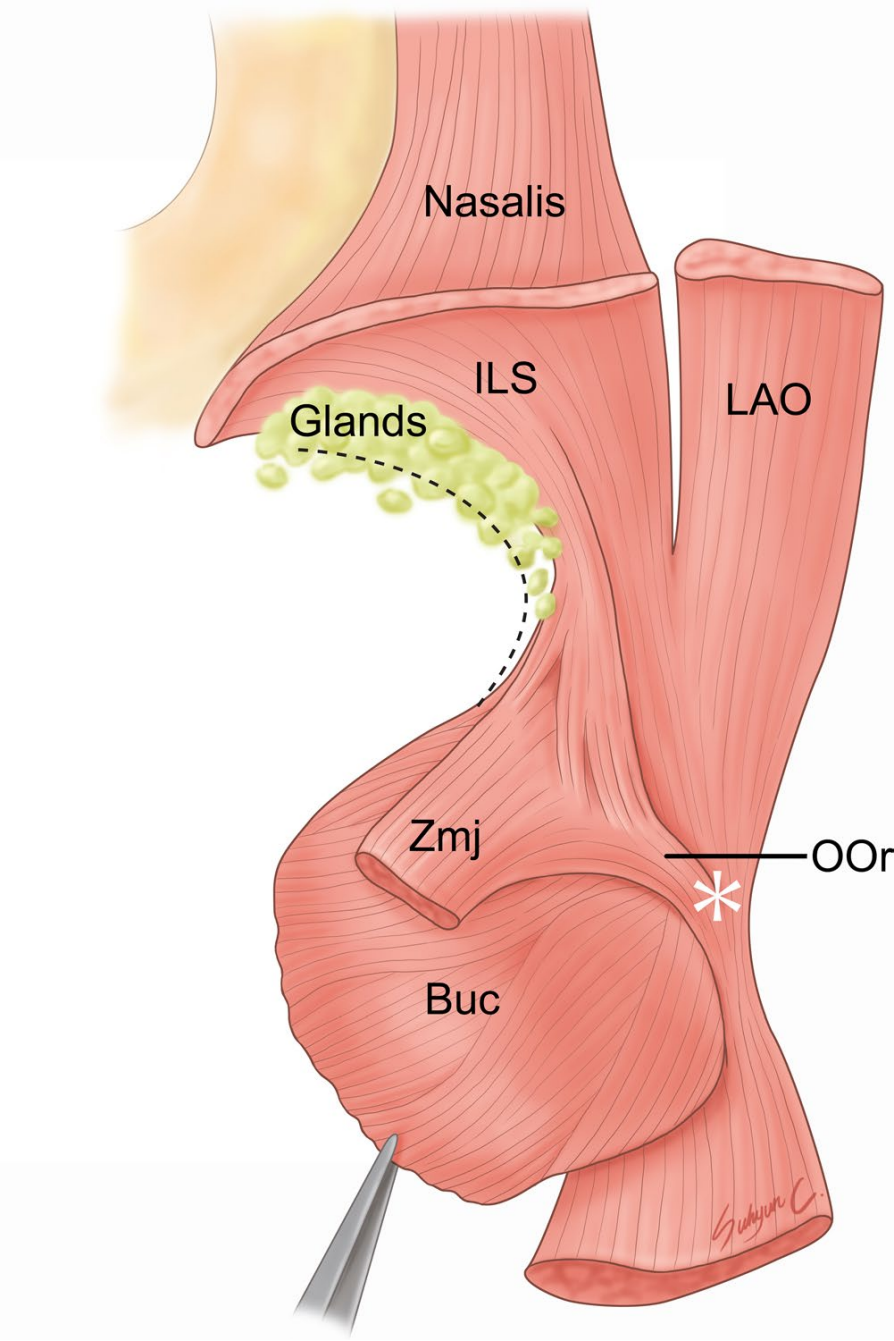
Schematic of the general pattern of the incisivus labii superioris muscle (ILS) in the posterior aspect. The ILS has an oblique and linear origin from the incisive fossa of the maxilla to the point just medial to the origin of the levator anguli oris muscle (LAO). The medial two-thirds of the arising fibers are more curved than the lateral one-third of the fibers. The arising fibers of the ILS arch and cover the prominent labial glands at the superior margin of the orbicularis oris muscle (OOr). After the ILS courses laterally along the anterior part of the upper mucolabial fold, it divides into the superficial and deep inserting fibers. The superficial inserting fibers blend with the medial fibers of the LAO to converge toward the modiolus, while the other fibers become deep inserting fibers that blend with the deep fibers of the OOr and zygomaticus major muscle (Zmj). Dashed line indicates the upper mucolabial fold. An asterisk indicates the modiolus. *Illustration licensed under CC BY 4*.*0*.

The ILS and ILI are attached to the incisive fossa of the maxilla and mandible, respectively, and they are the deepest fibers in the lip^4^. The ILS has a more complicated course than the ILI. The ILS courses along the convex superior peripheral part of the OOr, blending with several muscles in the modiolar area, while the ILI courses laterally and upward along the lower mucolabial fold and blends with the OOr or buccinator below the level of the corner of the mouth^5,11,12^. The present study has clearly demonstrated several anatomical features of the ILS (Fig. 9):

1. The ILS courses laterally along the anterior part of the upper mucolabial fold.
2. The arising ILS fibers arch and cover the prominent labial glands at the superior margin of the OOr.
3. The ILS fibers blend with several muscles in the modiolar area.
4. The ILS is located between the OOr and LAO in a continuous arrangement.

These observations indicate that the ILS may assist to compress the labial glands and the upper oral vestibule, controlling modiolar movements and thereby integrating the movements of the mouth and lips. In addition, the relationship of the mimetic muscles including the ILS with the oral mucosa can be important during performing oral surgery and dentistry^14^.

Iwanaga *et al*.^13^ described that the ILS consisted of two parts, inferior and superior, with the former merging into the OOr and the latter into the transverse and alar parts of the nasalis. Burkitt and Lightoller (1927)^15^ described that some of the lateral fibers of the ILS interlace with and have a common origin with those of the transverse parts of the nasalis in Aboriginal Australians. Other fibers interlace with the alar part of the nasalis, and the most-medial fibers are continuous with those fibers of the OOr that extend up to the septum. However, in the present study, some arising fibers of the ILS were intermingled with those of the transverse and alar parts of the nasalis at their origin sites. The ILS fibers were not merged with the nasalis nor attached to the septum, instead usually blending with the OOr and LAO in the modiolar area.

The ILS surrounded the anterior part of the upper mucolabial fold and corner of the mouth in the present study. In addition, the ILS deepened the superior margin of the OOr, thus increasing the convexity of the OOr and ILS. The convex shapes of the OOr and ILS surrounding the upper mucolabial fold and the corner of the mouth appeared to efficiently compress and control the oral vestibule and corner of the mouth, and may help to push food from the oral vestibule into the oral cavity proper.

Textbooks describe the ILS as originating from the incisive fossa of the maxilla^4,5,16^. Iwanaga *et al*.^13^ reported that the bony attachment of the ILS to the alveolar process had a triangular shape. However, in the present study it was found that the ILS had a linear origin from the incisive fossa to the point just medial to the origin of the LAO. Also, the ILS was located between the OOr and LAO in a fan shape. This continuous arrangement of the OOr, ILS, and LAO appears to make the upper lip and the corner of the mouth move successively, especially when the lips protrude.

The literature contains few descriptions about the relationships of the ILS with the labial glands. Burkitt and Lightoller (1926)^10^ described the ILS as being in contact with some mucous glands of the upper lip in Aboriginal Australians. The present study found that the arising fibers of the ILS arched and covered the prominent labial glands at the superior margin of the OOr. It is therefore thought that the ILS compresses the prominent labial glands at the superior margin of the OOr, while the OOr can compress other labial glands that were situated between the OOr and the mucous membrane.

Standring (2016)^5^ described that the ILS segregates into superficial and deep parts as it approaches the modiolus: the former blends partially with the LAO and attaches to the body and apex of the modiolus, while the latter is attached to the superior cornu and base of the modiolus. All of the superficial inserting fibers and some of the deep inserting fibers of the ILS converged toward the modiolus in the present study. A thin muscle layer that was formed by some fibers of the ILS and LAO converged toward the modiolus in 19.2% of the specimens. The superficial and deep inserting fibers of the ILS and the thin muscle layer coursed in different curved planes. Unlike other muscles that attach to the modiolus, the ILS can pull the modiolus superomedially. Thus, the several slips of the ILS may pull the modiolus superomedially in different planes. This arrangement can delicately control the three-dimensional mobility of the modiolus, enabling the fibers to integrate the activities of the oral vestibule so as to protrude the lips during drinking, sucking, swallowing, and speech.

There have been few descriptions about the attachment of the deep inserting fibers of the ILS to other muscles in the modiolar area. Some deep inserting fibers of the ILS blended with the lateral deep fibers of the OOr in most specimens of the present study. The fibers of the ILS blending with the lateral deep fibers of the OOr may therefore reinforce the OOr so as to assist closing of the mouth.

The ILS and ILI as well as the peripheral and marginal parts of the OOr are more differentiated in humans than in chimpanzees. Huber (1931)^8^ stated that the ILS and ILI have become more clearly differentiated from the OOr–buccinator system in humans than in chimpanzees and gorillas. Lightoller (1925)^17^ and Pellatt (1979)^18^ stated that the peripheral and marginal parts of the OOr are not clearly differentiated in chimpanzees. Contraction of the pars peripheralis fibers in humans positions the lip in labial elevation (an action involved in both facial expression and speech), while the pars marginalis may be regarded as being involved in speech modification^17,19^. Thus, the ILS and ILI in humans may reinforce the peripheral part of the OOr for more-delicate facial expressions, speech, and movements of the mouth and lips.

Iwanaga *et al*.^13^ reported that the lateral border of the inferior part of the ILS coincided with the upper buccal frenulum. They described that the ILS can be affected when performing an upper labial vestibule incision and correction of the gingival smile. In the present study the medial origin of the ILS was located in the periosteum adjacent to the upper mucolabial fold, which means that it may be cut during surgical procedures involving the labial vestibule. The muscle fibers of the peripheral part of the OOr are distorted and deviated in cleft lip^20–23^, while the correct arrangement of the muscle fibers is essential for successful primary repair of the cleft lip^23^. Therefore, the arrangement of the muscle fibers of the ILS and their relationships with other structures should be considered especially when performing vestibuloplasty and cleft lip repair.

In conclusion, this study has revealed several anatomical features of the ILS related to the labial glands, upper mucolabial fold, and modiolar area. The reported results will be helpful when analyzing the movements of the mouth and lips and when performing various types of facial surgery.

## Materials and Methods

All cadavers used in the present study were legally donated to Catholic Kwandong University College of Medicine. The present study was conducted in accordance with the Declaration of Helsinki. ILSs were investigated in 52 specimens obtained from formalin-fixed Korean adult cadavers (26 left sides, 26 right sides of the faces; 15 men, 11 women; mean age, 70.8 years; age range, 51–90 years). The muscles of the lower face were dissected and removed en bloc from the facial bones. The inner surfaces of the removed facial muscles were carefully dissected with the aid of a surgical microscope (OPMI-FC, Carl Zeiss, Oberkochen, Germany). The origin of the ILS was identified after cutting and folding the periosteum adjacent to the upper mucolabial fold, and then its fibers were followed to reveal its courses, connections, and attachments to surrounding structures. The most-lateral and most-medial points of the origin of the ILS were checked in the deep surface of the face, and then the corresponding points on the superficial surface of the face were determined by using piercing pins. The location of the ILS was observed on the superficial surface and in the deep surface of each facial muscle. All side-on photographs in this article are viewed from the right side of the face.

### Ethical approval

The methods were carried out in accordance with the 1964 Helsinki declaration and the cadavers were legally donated for the research by Catholic Kwandong University College of Medicine.

## Data Availability

The datasets generated during and/or analysed during the current study are available from the corresponding author on reasonable request.
